# Calculating disability-adjusted-life-years lost (DALYs) in discrete-time

**DOI:** 10.1186/1478-7547-11-18

**Published:** 2013-08-08

**Authors:** Bruce A Larson

**Affiliations:** 1 Department of International Health, Boston University School of Public Health, 801 Massachusetts Avenue, Boston, MA 02118, USA; 2 Center for Global Health and Development, Boston University, 801 Massachusetts Avenue, Boston, MA 02118, USA

**Keywords:** Disability adjusted life years lost, Discrete time, Cost-effectiveness

## Abstract

Disability-adjusted-life-years lost (DALYs) is a common outcome metric for cost-effectiveness analyses, and the equations used for such calculations have been presented previously by Fox-Rushby and Hanson (see, e.g., “Health Policy and Planning 16:326–331, 2001”). While the equations are clear, the logic behind them is opaque at best for a large share of public health practitioners and students. The objective of this paper is to show how to calculate DALYs using a discrete time formulation that is easy to teach to students and public health practitioners, is easy to apply for those with basic discounting skills, and is consistent with the discounting methods typically included on the costing side of cost-effectiveness analysis. A continuous-time adjustment factor is derived that can be used to ensure exact consistency between the continuous and discrete time approaches, but this level of precision is typically unnecessary for cost-effectiveness analyses. To illustrate the approach, both a new, simple example and the same example presented in Fox-Rushby and Hanson are used throughout the paper.

## Background

The concept of disability adjusted life years lost (DALYs) was introduced in the 1993 World Development Report as a way to estimate and compare the burden of morbidity and premature mortality caused by widely varying conditions and states within and among countries [1]. DALYs are calculated as the present discounted value of future years of healthy life lost to morbidity/disability and future years of life lost to premature mortality [2,3]. Building on this earlier literature, Fox-Rushby and Hanson, hereafter referred to as FRH, provided a clear standard for calculating DALYs as an outcome indicator for cost-effectiveness analyses [4]. FRH provide the rather arduous equations for years of life lost (YLL) and years lived disabled (YLD), which are an artifact of the continuous-time math used in the development of DALYs. Over the past twenty years, a wide range of analyses in developed and developing countries have used DALYs in cost-effectiveness analyses [5-13].

Calculating the present discounted value of a stream of future costs or benefits is a standard topic in most courses that include economic evaluation and cost-effectiveness. Time is typically measured in discrete units (months, years, etc.), and present values are calculated easily in a spreadsheet program. Discrete time units are also typically the denominator in reported economic and health data (e.g. annual program costs, annual mortality figures, and so on). While useful in most other ways, however, discrete time units are not consistent with the traditional calculation of DALYs with time as a continuous variable [14].

The purpose of this paper is to present a very simple approach for calculating DALYs in discrete time for cost-effectiveness analyses. Calculating DALYs using a discrete time formulation is easy to implement in a spreadsheet program and provides sufficient precision for most users’ purposes. This approach is easy to teach to students and public health practitioners, is easy to apply for those with basic discounting skills, is consistent with the discounting methods typically included in the costing side of cost-effectiveness analysis, and all the assumptions imbedded in the analysis are very transparent. The approach is applied to a new simple example as well as to the same worked-out example provided in FRH [4].

### The discrete-time approach

The costing component of cost-effectiveness analyses typically uses standard, discrete-time discounting in the calculation of the present value of program costs and/or the annual equivalent costs for multi-year programs [15]. With time in years, the discrete time discount factor, 1/(1+r)^t^, where r is the discount rate and t the number of years, is commonly used.

The following simple example will be used throughout the paper. Suppose a man dies on his 55^th^ birthday, and age-specific additional life expectancy for this age was 10 years in his country. Without discounting, 10 years of life are lost, so YLL(0, 0) = 10.

With 10 years of life lost and a discount rate of 3%, Table 1 shows how a spreadsheet is typically organized to calculate the present discounted value of a stream of numbers (e.g., costs, payments, or in this case years of life lost). In Table 1, the ‘present’ is time 0, meaning the time at which the evaluation is being completed. Having time progress from 0 to 9 assumes that each of the 10 years lost is counted at the beginning of the year, which is the same as stream of payments where each payment is made at the beginning of the year. Using this calculation for the 10 years of life lost, YLL(3, 0) = 8.786 with discrete time discounting.

**Table 1:** **Calculating YLL(3, 0) for a person who dies on their 55**^**th **^**birthday with age-specific life expectancy of 10 years with annual discounting as 1/(1+r)**^**t**^

**Which year**	**t**	**Year**	**Discrete time**	**Discounted**
**was lost**		**lost**	**discount factor**	**year lost**
		A	B = 1/(1 + 0.03)^t^	C = A*B
56	0	1	1.000	1.000
57	1	1	0.971	0.971
58	2	1	0.943	0.943
59	3	1	0.915	0.915
60	4	1	0.888	0.888
61	5	1	0.863	0.863
62	6	1	0.837	0.837
63	7	1	0.813	0.813
64	8	1	0.789	0.789
65	9	1	0.766	0.766
	Sum of	10	Sum of discounted	8.786
	years lost		year lost column	

How does this answer compare to the continuous-time approach? In the absence of age weighting, the equation for years of life lost due to premature mortality provided in FRH reduces to:

(1) $$\mathrm{YLL}\left(r,0\right)=\int_{0}^{L}1*e^{-\mathrm{rt}}\mathrm{dt}=\left(\frac{1}{r}\right)\;\left(1-e^{-\mathrm{rL}}\right)$$

where 0 is the ‘present’ , L is age-specific life expectancy for the person who died prematurely, representing the years of life lost for the person who died, and r is the discount rate. For notational simplicity YLL(r, 0) is used for years of life lost in Equation (1). With a 3% discount rate and 10 years of life expectancy, the answer to Equation (1) is 8.6394.

The basic point here is that the 1.7% difference between a simple discrete-time approach and the continuous time approach is minor, and such differences will not change fundamentally any ranking in a cost-effectiveness analysis. In addition, anyone who has actually completed the costing component of a cost-effectiveness analysis knows how many assumptions, estimates, and outright guesses are included in the cost analysis. The age-specific life expectancy figures inherent in DALY calculations similarly rely on data of varying quality, and the same kinds of uncertainties affect estimates of the change in DALYs due to an intervention or policy change as well.

### The FRH DALY(3, 0) example using this simple approach

Another benefit of the simple approach is that a more complicated scenario can easily be incorporated into the same spreadsheet, no equations needed. FRH consider a woman who develops bipolar depression at age 35, lives for 10 years with the disorder, and then dies prematurely at age 45. The disability weight is 0.60, life expectancy at age 45 is 34.73 years (age 79.73), and the “present” for the present value calculation is age 35 (meaning exactly on her 35^th^ birthday).

Table 2 shows the spreadsheet used to calculate DALY(3, 0) for this example without age weighting (see the appendix for age weighting). From time 0 through time 9, representing her 36^th^- 45^th^ years of life, she loses 0.6 years of life annually. From time 10 through 43, representing her 46^th^ – 79^th^ years of life, she loses 1 year of life annually. In time 44 (80^th^ year of life), she loses 0.73 years of life. Using the discrete time discount factor 1/(1+r)^t^, the present value of the stream of lost years of life (0.60 per year when disabled plus 1.00 per year when deceased) is 21.666.

**Table 2:** **Calculating DALY(3, 0) for a women who develops bipolar disorder on 35th birthday, lives 10 years, and life-expectancy at age 45 is 34.73 years, with annual discounting as 1/(1 + r)**^**t**^

**Which**	**t**	**Year lost (either**	**Discrete time**	**Discounted**
**year**		**disability weight**	**discount**	**year lost**
**was lost**		**or death)**	**factor**	
36	0	0.6	1.000	0.600
37	1	0.6	0.971	0.583
38	2	0.6	0.943	0.566
39	3	0.6	0.915	0.549
40	4	0.6	0.888	0.533
41	5	0.6	0.863	0.518
42	6	0.6	0.837	0.502
43	7	0.6	0.813	0.488
44	8	0.6	0.789	0.474
45	9	0.6	0.766	0.460
46	10	1	0.744	0.744
47	11	1	0.722	0.722
48	12	1	0.701	0.701
49	13	1	0.681	0.681
50	14	1	0.661	0.661
51	15	1	0.642	0.642
52	16	1	0.623	0.623
53	17	1	0.605	0.605
54	18	1	0.587	0.587
55	19	1	0.570	0.570
56	20	1	0.554	0.554
57	21	1	0.538	0.538
58	22	1	0.522	0.522
59	23	1	0.507	0.507
60	24	1	0.492	0.492
61	25	1	0.478	0.478
62	26	1	0.464	0.464
63	27	1	0.450	0.450
64	28	1	0.437	0.437
65	29	1	0.424	0.424
66	30	1	0.412	0.412
67	31	1	0.400	0.400
68	32	1	0.388	0.388
69	33	1	0.377	0.377
70	34	1	0.366	0.366
71	35	1	0.355	0.355
72	36	1	0.345	0.345
73	37	1	0.335	0.335
74	38	1	0.325	0.325
75	39	1	0.316	0.316
76	40	1	0.307	0.307
77	41	1	0.298	0.298
78	42	1	0.289	0.289
79	43	1	0.281	0.281
80	44	0.73	0.272	0.199
	Sum of	40.73	Sum of	21.666
	years		discounted	
	lost		year lost	
			column	

How does the answer from this simple approach compare to the FRH answer? For the 10 years lived disabled, we have:

(2) $$\mathrm{YLD}\left(3,0\right)=\;0.6\left(\frac{1}{0.03}\right)\;\left(1-e^{-0.03*10}\right)=\;5.1836$$

For the 34.73 of life lost, we have:

(3) $$\mathrm{YLL}\left(3,0\right)=\;e^{-0.03*10}\left(\frac{1}{0.03}\right)\;\left(1-e^{-0.03*34.73}\right)=\;15.9823$$

Combining the answers in Equation (2) and (3), DALY(3, 0) using the continuous time approach is 21.166. Thus, the simple discrete time approach yields a result that is 2.4% higher than the FHR answer.

### A minor adjustment for discounting in discrete time

The continuous-time approach embedded into standard DALY calculations uses e^-rt^ as the discount factor rather than 1/(1+r)^t^. In practice, e^-rt^ is a little less than 1/(1+r)^t^, so that present discounted values will be slightly different depending on which formulation of the discount factor is used. If r = 0.03 is the discount rate when discounting using e^-0.03t^, then a discount rate for discrete time of r^d^ = e^0.03^ – 1 = 0.03045453 will yield the identical discount factor (this point is also explained in [2]). This slightly higher discount rate, for example, reduces slightly the estimated DALYs lost in Table 2 to 21.485, which is 1.5% higher than the FRH answer.

### An exact adjustment for the discrete approach

In the absence of age weighting, the simple discrete approach, perhaps using e^-rt^ for annual discounting, is more than adequate for program evaluations and applied cost-effectiveness analyses. If, however, an analyst feels compelled to provide the ‘exact’ FRH answer, one simple adjustment can be made.

With the commonly assumed 3% discount rate, one year of life is lost is:

(4) $$\mathrm{YLL}\left(3,0\right)=\;\left(\frac{1}{0.03}\right)\;\left(1-e^{-0.03}\right)=\;0.985148881716395$$

In other words, one year of life lost is less than one because the year is lost continuously over the full year, instead of being counted all at once at the beginning for the year (as in Table 1 and Table 2).

Table 3 replicates the analysis in Table 1 with the addition of this continuous time adjustment factor (0.985148881716395). In Table 1, each year of life lost is multiplied by this adjustment factor, and then discounted using the e^-rt^ formulation. With this addition to the analysis, YLL(3, 0) = 8.6394, which is the exact FRH answer.

**Table 3:** **Calculating YLL(3, 0) for a person who dies on their 55th birthday with age-specific life expectancy of 10 years, annual discounting as e**^**-rt **^**, and the continuous time adjustment factor**

**t**	**Year**	**Continuous**	**Adjusted**	**Discrete**	**Discounted**
	**lost**	**time**	**year lost**	**time**	**and**
		**adjustment**	**for**	**discount**	**adjusted**
		**factor**	**continuous**	**factor**	**year lost**
			**time**		
	A	B	C = A*B	D = e^-rt^	E = C*D
0	1	0.9851	0.985148882	1.000	0.9851
1	1	0.9851	0.985148882	0.970	0.9560
2	1	0.9851	0.985148882	0.942	0.9278
3	1	0.9851	0.985148882	0.914	0.9004
4	1	0.9851	0.985148882	0.887	0.8737
5	1	0.9851	0.985148882	0.861	0.8479
6	1	0.9851	0.985148882	0.835	0.8229
7	1	0.9851	0.985148882	0.811	0.7985
8	1	0.9851	0.985148882	0.787	0.7749
9	1	0.9851	0.985148882	0.763	0.7520
Sum	10			Sum of	8.6394
of				discountedt	
years				and adjusted	
lost				years lost	

The analysis in Table 2 can also be replicated using the same approach; simply multiply the year lost or lived disabled by the continuous time adjustment factor and use e^-rt^ for discounting. The answer is DALY(3, 0) = 21.165 while the FRH approach yields 21.166. The reason for the minor discrepancy is that the final 0.73 of a year is simply weighted by the annual continuous time adjustment factor. To be exactly correct for that last 0.73 of a year, Equation (1) using L = 0.73 equals 0.7221. If 0.7221 is used instead of 0.73, the result becomes 21.166.

In summary, the discrete approach outlined above yields the same answer as the continuous time approach using e^-rt^ as the discount factor and adjusting each year of death or morbidity/disability by the continuous time adjustment factor (from Equation 4).

## Discussion

While disability adjusted life years have traditionally been calculated using the continuous time formulation reviewed presented in FRH, a simple approach using discrete time to calculate the present discounted value is easy to implement, easy to explain to practitioners and policy makers, and consistent with methods typically used on the costing side of cost effectiveness analysis.

## Appendix

What about age weighting in discrete time?

The basic age-weighting function used in FRH and elsewhere is:

(5) $$a\left(x\right)=\mathrm{Cx}e^{-\mathrm{\beta x}}=0.1658xe^{-0.04x}$$

In general, this same age weighting function can be used in discrete time. Equation (5) implies, however, an age weight of 0 during the first year of life (birth through 1^st^ birthday), even though in continuous time a(x) grows from 0 at birth to 0.159 at one year.

To allow for the first year of life to have a positive age weight, the basic age weighting function can be adjusted to the mid-point for each year, so that:

(6) $$\begin{array}{rl} a\left(x\right) & =C\left(x+0.5\right)e^{-\beta \left(x+0.5\right)}\\ & =0.1658\left(x+0.5\right)e^{-0.04\left(x+0.5\right)} \end{array}$$

This formulation allows the first year of life in discrete time to have a positive age weight (0.08126), and this age weight for the 1^st^ year of life is identical (to 5 decimal places) to the integral of (5) evaluated over 0 to 1.

For the future, the logic of using 0.1658 in the age-weighting function for cost-effectiveness analyses, is not especially solid. With β = 0.04, C=0.1658 was chosen so that global DALYs were the same with and without age weighting in the 1990 global burden of disease study [16]. With the new 2010 GBD estimates, the future of C=0.1658 seems limited.

As an alternate, the approach suggested in the 2006 GDB report for values of β other than 0.04 can be followed. In short, the value of C can be chosen so that total years added up to 100 when age weighted or not [16]. Using this same logic in discrete time, Table 4 shows a spreadsheet organized to identify C with β = 0.04. Once the age weight is included in the spreadsheet for any value of C, the value of C can be adjusted until the sum of the column over 100 years adds up to 100 (C = 0.176116). The age weights from Table 4 can be used to add age weighting into DALY calculations (if age weighting is included in the analysis).

**Table 4:** Age weights for discrete time

**C**	**0.176116**
β	0.04
Sum of Age Weights	100.000
Age (birth and future birthdays):	Age weight function:
a(x)	a(X) = C(x + 0.5)e^-β(X+0.5)^
0	0.086
1	0.249
2	0.398
3	0.536
4	0.662
5	0.777
6	0.883
7	0.979
8	1.066
9	1.144
10	1.215
11	1.279
12	1.335
13	1.386
14	1.430
15	1.468
16	1.502
17	1.530
18	1.555
19	1.574
20	1.590
21	1.602
22	1.611
23	1.617
24	1.619
25	1.619
26	1.617
27	1.612
28	1.605
29	1.596
30	1.586
31	1.574
32	1.560
33	1.545
34	1.529
35	1.511
36	1.493
37	1.474
38	1.454
39	1.433
40	1.412
41	1.390
42	1.367
43	1.345
44	1.322
45	1.298
46	1.275
47	1.251
48	1.227
49	1.204
50	1.180
51	1.156
52	1.132
53	1.109
54	1.085
55	1.062
56	1.038
57	1.015
58	0.992
59	0.970
60	0.947
61	0.925
62	0.904
63	0.882
64	0.861
65	0.840
66	0.819
67	0.799
68	0.779
69	0.759
70	0.740
71	0.721
72	0.703
73	0.684
74	0.666
75	0.649
76	0.632
77	0.615
78	0.598
79	0.582
80	0.566
81	0.551
82	0.536
83	0.521
84	0.507
85	0.493
86	0.479
87	0.465
88	0.452
89	0.439
90	0.427
91	0.415
92	0.403
93	0.391
94	0.380
95	0.369
96	0.358
97	0.348
98	0.337
99	0.327

## Abbreviations

DALY: Disability adjusted life year lost.

## Competing interests

The author declares that he have no competing interest.
